# Quantitative analysis on photon numbers received per cell for triggering β-carotene accumulation in *Dunaliella salina*

**DOI:** 10.1186/s40643-021-00457-4

**Published:** 2021-10-21

**Authors:** Yimei Xi, Song Xue, Xupeng Cao, Zhanyou Chi, Jinghan Wang

**Affiliations:** 1 Key Laboratory of Industrial Ecology and Environmental Engineering (Ministry of Education, China), School of Environmental Science and Technology, Dalian University of Technology, Dalian, 116024 China; 2 School of Bioengineering, Dalian University of Technology, Dalian, 116024 China; 3 Dalian Institute of Chemical Physics, Chinese Academy of Sciences, Dalian, 16023 China

**Keywords:** β-carotene, Average irradiance, *D. salina*, Photons received per cell

## Abstract

**Abstract:**

Accumulation of β-carotene in *Dunaliella salina* is highly dependent on light exposure intensity and duration, but quantitative analysis on photon numbers received per cell for triggering β-carotene accumulation is not available so far. In this study, experiment results showed that significant β-carotene accumulation occurred after at least 8 h illumination at 400 µmol photons·m^−2^·s^−1^. To quantify the average number of photons received per cell, correlations of light attenuation with light path, biomass concentration, and β-carotene content were, respectively, established using both Lambert–Beer and Cornet models, and the latter provided better simulation. Using Cornet model, average number of photons received per cell (APRPC) was calculated and proposed as a parameter for β-carotene accumulation, and constant APRPC was maintained by adjusting average irradiance based on cell concentration and carotenoids content changes during the whole induction period. It was found that once APRPC reached 0.7 µmol photons cell^−1^, β-carotene accumulation was triggered, and it was saturated at 9.9 µmol photons cell^−1^. This study showed that APRPC can be used as an important parameter to precisely simulate and control β-carotene production by *D. salina*.

**Graphic Abstract:**

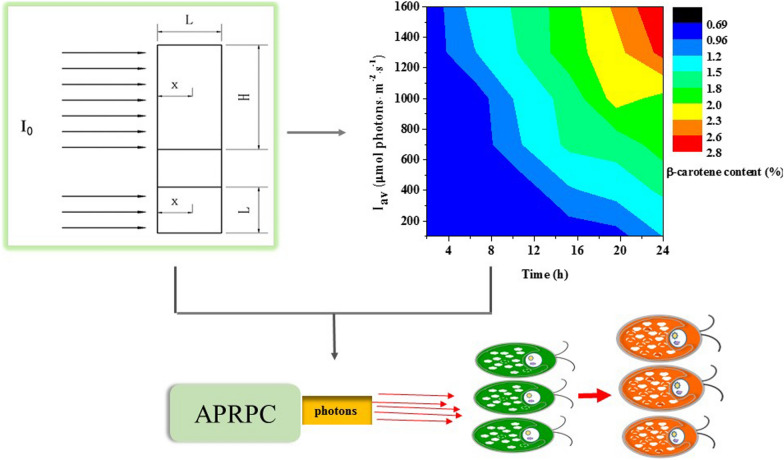

**Supplementary Information:**

The online version contains supplementary material available at 10.1186/s40643-021-00457-4.

## Introduction

β-carotene has wide applications in the nutraceuticals, cosmetics, and food industries, and its current global consumption is about 1000 tons per year (Gong et al. 2016). There has been a rising demand for natural β-carotene, instead of synthetic products, due to the advantages of its mixed stereoisomers of all-*trans* and 9-*cis* β-carotene, which are more fat-soluble than synthetic β-carotene (Combe et al. 2015; Ben-Amotz and Avron 1989). Microalgae can serve as promising feedstock for natural β-carotene production, among which, green alga *Dunaliella salina* has been regarded as the best candidate because of its high β-carotene content (up to 14%) (Borowitzka et al. 1988; Fachet et al. 2016; Rabbani et al. 1998). The major function of the β-carotene accumulation in *Dunaliella* is energy dissipation and photo protection, which turn excess energy into heat (Gong et al. 2016; Combe et al. 2015).

For indoor production of β-carotene from *Dunaliella*, a two-stage cultivation strategy is usually applied for β-carotene accumulation (Liang et al. 2019). In the first stage, also known as the 'green stage', nutrient replete medium and optimal light intensity are adopted to obtain the green vegetative cells. Then in the second stage (also refer to as the ‘red stage’ or ‘induction stage’), green cells are subjected to various stress conditions such as high irradiance, high salinity and nitrate/phosphate deprivation to stimulate β-carotene accumulation (Ben-Amotz et al. 1987; Kleinegris et al. 2011a, b; Lamers et al. 2012; Masojidek et al. 2009; Pereira et al. 2019). Up till now, such stress-induced *D. salina* β-carotene accumulation is mostly qualitatively investigated (Phadwal et al. 2003; Gong et al. 2016; Fachet et al. 2016; Bonnefond et al. 2017), while the exact quantitative relationship between stress and β-carotene accumulation is so far unknown. Among various stresses, light irradiance is considered to be the most important environmental parameter for β-carotene induction (Lamers et al. 2010). Many efforts have been made on the coordination between incident light intensity and carotenoids accumulation in *D. salina* (Fachet et al. 2016; Gomez and Gonzalez 2005; Wu et al. 2016; Xu et al. 2016). However, the local light intensity decayed exponentially with the increase of light path and since light penetration decreases with cell growth and β-carotene accumulation, the relationship between initial light intensity at the culture medium surface and final β-carotene content in *D. salina* cells is ambiguous, thus cannot be applied for real-world β-carotene production guidance, which unfortunately takes place in various cultivation configurations and at different culturing densities. Quantifying photon numbers received per cell to initiate β-carotene accumulation, therefore, is necessary for establishing a meaningful relationship between illumination and *D. salina* β-carotene production.

Clarifying average irradiance (number of photons) reaching each cell in the culture is the prerequisite to realize the above goal. According to previous studies, incident light intensity, microalgae cell density, light path, and light extinction coefficient all have influences on the average irradiance for microalgae (Richmond et al. 1999, 2003; Yun et al. 2001; Huang et al. 2014; Mchardy et al. 2018), whereas the correlation between light extinction coefficient and average irradiance is complicated by pigment accumulation (especially β-carotene) during the induction stage (Bechet et al. 2013; Fernandez et al. 1997; Martinez et al. 2012). Although the influence of pigment content on light extinction coefficient is available for other microalgal strains (e.g., astaxanthin in *Haematococcus pluvialis*) (Martinez et al. 2012; Gao et al. 2017), little effort has been focused on the impact of β-carotene content on light attenuation during β-carotene accumulation in *D. salina* suspension, which therefore requires in-depth investigation.

The objective of this study is to quantify the effect of light availability on β-carotene accumulation. Firstly, light distribution in the *D. salina* suspension during β-carotene accumulation stage was simulated using Lambert–Beer model and Cornet model, then the complex relationship between β-carotene accumulation and light absorption coefficient and scattering coefficient was comprehensively investigated. The present study quantified “light stress” via a physical variable, namely the average number of photons received per cell (APRPC), and the APRPC was controlled through adjusting average irradiance corresponding to increased cell concentration and carotenoids content during the whole induction period. Critical values of APRPC for high β-carotene accumulation in *D. salina* were identified. Results of this study would be of reference value on average irradiance modeling and culture condition optimization, and could facilitate indoor and outdoor massive *D. salina* β-carotene production.

## Materials and methods

### Strain and medium

*Dunaliella salina* CCAP 19/18 was purchased from Culture Collection of Algae and Protozoa (Windermere, United Kingdom). The strain was previously maintained in modified Artificial Sea Water (ASW), with composition of 1.5 M NaCl, 5 mM KNO_3_, 0.45 mM MgCl_2_·6H_2_O, 0.05 mM MgSO_4_·7H_2_O, 0.3 mM CaCl_2_·2H_2_O, 0.13 mM K_2_HPO_4_, 0.02 mM FeCl_3_, 0.02 mM EDTA, and 1 mL of trace elements stock containing 50 mM H_3_BO_3_, 10 mM MnCl_2_·4H_2_O, 0.8 mM ZnSO_4_·7H_2_O, 0.8 mM CuSO_4_·5H_2_O, 2 mM NaMoO_4_·2H_2_O, 1.5 mM NaVO_3_, and 0.2 mM CoCl_2_·6H_2_O, and the pH was adjusted to 7.5 by adding 40 mM of Tris-buffer (Doddaiah et al. 2013). Stock culture was performed in 500-mL conical flasks at 50 µmol photons·m^−2^·s^−1^ light intensity. After reaching steady state, the microalgal biomass was inoculated into a set of multi-device-equipped flat-plate photobioreactors (PBRs) named “Algal Station” (Cao et al. 2019), and a schematic diagram of the system is displayed in Fig. S1.

### Cultivation condition

Using the “Algal Station”, incident light intensities on the PBR surface by white LEDs were feedback controlled according to the measured outgoing irradiance, biomass concentration, and β-carotene content during the whole induction period. Incident light intensities and transmitted light intensities were recorded online by NHGH09 photosynthetically active radiation sensor (Wuhan Zhongke Nenghui Technology Development Co.,Ltd)*.* Light paths of the PBRs were, respectively, 0.025 m, 0.05 m and 0.10 m. The cultivation temperature was automatically controlled at 25 ± 0.5 °C, while pH level was maintained at 7.5 ± 0.2 by pulsing CO_2_ mixed with air. Cultures in the PBR were agitated with 0.2 µm membrane filtered air at 400 mL·min^−1^.

### Experimental design

#### Light transfer modeling in PBRs

To model the light distribution inside the culture, a set of experiments were conducted at incident light intensity 400 µmol photons·m^−2^·s^−1^ with various biomass concentrations (0.10, 0.25, 0.50, 0.75, 1.0 and 1.5 g·L^−1^ DW) and light paths (0.025 m, 0.05 m and 0.10 m). Local light intensity in the culture, along with biomass concentration, were measured at different light paths using the NHGH09 photosynthetically active radiation sensor. This data was used to model light attenuation as a function of biomass concentration and light path, the measurements were carried out in triplicate.

In order to investigate the effect of β-carotene content on the light attenuation at different microalgae concentration, low biomass concentration (0.5 g·L^−1^) and high biomass concentration (1.5 g·L^−1^) were chosen, and the cell density was adjusted to the required cell density after culture the microalgae. The Lambert–Beer model and Cornet model light transfer models were employed to calculate the local light intensity at different light paths (0.025, 0.05, 0.10 m) and five different β-carotene contents (0.56%, 0.84%, 1.21%, 1.93%, 2.88%, 4.26% DW).

#### APRPC calculation

The average number of photons received per cell was calculated, termed as APRPC, calculated by Eq. (1):

1 $$ {\text{APRPC }} = \frac{{I_{{{\text{av}}}} \cdot T \cdot S \cdot L \cdot 1000}}{C}, $$

where *APRPC* is average number of photons received per cell (μmol photons·cell^−1^), *I*_*av*_ is the averaged irradiance (μmol photons·m^−2^·s^−1^), *T* is the induction time (s), which is the time length of cultures under light exposure, starting from the time that the cells were transferred into the induction PBR. *S* is the area of illumination surface (0.051 m^2^), *L* is light path (0.025 m) and *C* is cell number (cell·L^−1^).

#### Short-term effect of APRPC on β-carotene accumulation

*D. salina* were cultivated for different time lengths (2, 4, 8, 12, 16, 24 h) and under different average irradiances (100, 200, 400, 800, 1200 and 1600 µmol photons·m^−2^·s^−1^), each treatment was repeated three times. In this experiment, the average irradiance inside the PBRs was kept constant through adjusting incident light intensity with increased cell concentration and carotenoids content during the whole induction period by “Algal Station” (Cao et al. 2019), and the ratio of incident irradiance and biomass concentration was used to optimize APRPC in batch cultures, thus APRPC could be controlled during the β-carotene accumulation induction stage. Pigment content and biomass measurements were conducted within 24 h of sampling. In general, when β-carotene content was 2 times higher than the initial content, it was regarded as significant β-carotene accumulation.

#### Long-term effect of APRPC on β-carotene accumulation

In order to get higher β-carotene content, long-term effect of APRPC on β-carotene accumulation in *D. salina* was investigated. A set of experiments were conducted at various average irradiance levels (50, 100, 400, 800, and 1200 µmol photons·m^−2^·s^−1^) and induction times (24, 48, 72, 96, 120, 144 h), the measurements were carried out in triplicate.

### Analytical methods

#### Dry weight determination

Dry weight was determined using pre-weighed Whatman GF/C filters (47 mm diameter). 10-mL cultures were filtered and washed three times with 2 mL 0.5 M ammonium bicarbonate (Zhu et al. 1997) and then dried at 60 °C for over 16 h until the weight was constant, dry weight (g∙L^−1^) of the microalgae cells was calculated by subtracting the clear filter weight from the final weight (Chi et al. 2016).

#### Pigment measurement

For determining the amount of pigments including chlorophyll (Chla and Chlb) and carotenoids, about 10 mg of dried biomass was extracted with 1 mL 90% (v/v) acetone, vortexed for 20 s, and then centrifuged at 10,000 rpm for 2 min. The above pigment extraction procedure was repeated until the solution was colorless. The absorbance of Chla, Chlb and carotenoids content was measured at 665, 645, and 470 nm, respectively, using a UV/VIS spectrophotometer (Jasco V-530, JASCO Corporation, Japan), according to the modified method of our previous study (Xi et al. 2020), and were calculated using the equations below:

2 $$ C_{{{\text{Chla}}}} \left( {{\text{mg}} \cdot {\text{L}}^{{ - {1}}} } \right) = 11.75 \, \left( {A_{665} } \right) - 2.35 \, (A_{645} ), $$

3 $$ C_{{{\text{Chlb}}}} \left( {{\text{mg}} \cdot {\text{L}}^{{ - {1}}} } \right) = 18.61 \, \left( {A_{645} } \right) - 3.96(A_{665} ), $$

4 $$ {\text{Total carotenoids}}\left( {{\text{mg}} \cdot {\text{L}}^{{ - {1}}} } \right) = \left( {1000A_{470} - 2.270 \, C_{Chla} - 81.4 \, C_{Chlb} } \right)/198, $$

where *C*: pigment concentration (mg∙L^−1^), *A*_*x*_ is absorbance at x nm wavelength.

5 $$ {\text{Pigment content}}\left( \% \right) = \frac{{{\text{Pigment}}\;{\text{concentration}}\;({\text{mg}} \cdot {\text{L}}^{ - 1} ) \times {\text{volumn}}\;(5\,{\text{mL}})}}{{M\,({\text{mg}})}} \times 0.001 \times 100\% $$

where *M*: dry cell weight (mg).

A modified spectrophotometric method was used to determine β-carotene content in the biomass (Zhu et al. 2018). 1 mL of cell suspension was centrifuged at 10,000 rpm for 2 min. After centrifugation, the supernatant was discarded and 3 mL dodecane was added. The sample was shaken vigorously to re-suspend the algae pellets. Then, 9 mL of methanol was added to completely break up the cells and the tube was shaken vigorously again, then centrifuged for 2 min at 10,000 rpm. The dodecane-containing lipophilic carotenoids (upper layer) were measured with a spectrophotometer (Jasco V-530, JASCO Corporation, Japan) at 453 nm and 665 nm with dodecane as reference. β-carotene concentration was calculated as Eq. (6):

6 $$ C_{\beta - car} \left( {{\text{mg}} \cdot {\text{L}}^{{ - {1}}} } \right) = \, (A_{453} - \, A_{665} /3.91) \times 3.657 \times 3 \times X, $$

where *(A*_*453*_* − A*_*665*_*/3.91*) is the absorbance of β-carotene corrected for chlorophyll contamination, 3.657 is the calibration factor derived from HPLC analysis of β-carotene concentration, 3 is the amount of milliliters of dodecane added for extraction, and *X* is the dilution factor to measure absorbance on spectrophotometer (Kleinegris 2011a, b).

The amount of β-carotene in the algae biomass was calculated according to Eq. (7):

7 $$ \beta - {\text{carotene }}\left( \% \right) \, = \frac{{C_{{\beta - {\text{car}}}} \times 10}}{DW} $$

where *C*_*β-car*_ is the β-carotene content (mg∙L^−1^), *DW* is the cell dry weight (g·L^−1^).

### Light distribution model establishment

#### Light attenuation analysis

Two models were adopted to analyze light attenuation inside the microalgae suspension, i.e., Lambert–Beer model (Eq. (8)) (Bechet et al. 2013), and Cornet model (Eqs. (9–11)) (Fernandez et al. 1997).

8 $$ I = I_{0} \cdot e^{{( - L \cdot (K_{a} \cdot X + b))}} , $$

where *I* is the local light intensity (μmol photons·m^−2^·s^−1^), *I*_*0*_ is the incident light intensity (µmol·photons·m^−2^·s^−1^), *K*_*a*_ is the extinction coefficient (m^2^·g^−1^), *X* is the microalgae concentration (g·L^−1^), *b* is the fitting constant (m^−1^) and *L* is the light path (m):

9 $$ \frac{I}{{I_{0} }} = \frac{{4\alpha_{1} }}{{(1 + \alpha_{1} )^{2} \cdot e^{{\alpha_{2} }} - (1 - \alpha_{1} )^{2} \cdot e^{{ - \alpha_{2} }} }}, $$

10 $$ \alpha_{1} = \sqrt {\frac{{E_{a} }}{{E_{a} + E_{s} }}} , $$

11 $$ \alpha_{2} = (E_{a} + E_{s} ) \cdot \alpha_{1} \cdot X \cdot L, $$

where *E*_*a*_ is the mass absorption coefficient (m^2^·g^−1^), and *E*_*s*_ is the mass scattering coefficient (m^2^·g^−1^), *L* is the light path (m), and *X* is the microalgae concentration (g·L^−1^). *α*_*1*_ and *α*_*2*_ represent the correlation between *E*_*a*_ and *E*_*s*_. Matlab 2014 was employed to estimate the parameters of Lambert–Beer model and Cornet model.

#### Average irradiance calculation

In the flat-plate reactor, the average light intensity can be calculated as Eq. (12) (Suh and Lee, 2001):

12 $$ I_{av} = {\raise0.7ex\hbox{$1$} \!\mathord{\left/ {\vphantom {1 V}}\right.\kern-\nulldelimiterspace} \!\lower0.7ex\hbox{$V$}} \cdot \int_{0}^{V} {I{\text{d}}v} , $$

where *I*_*av*_ is the volume-averaged irradiance (μmol photons·m^−2^·s^−1^), *V* is the volume of the PBR (L), and *I* is the local light intensity (μmol photons·m^−2^·s^−1^).

### Statistical analysis

The one-way ANOVA analysis were performed in Excel (version 2013, Microsoft) to make a significance analysis for the β-carotene content and light intensity.

## Results

### Light attenuation in *D. salina* cell suspension

**Fig. 1:**
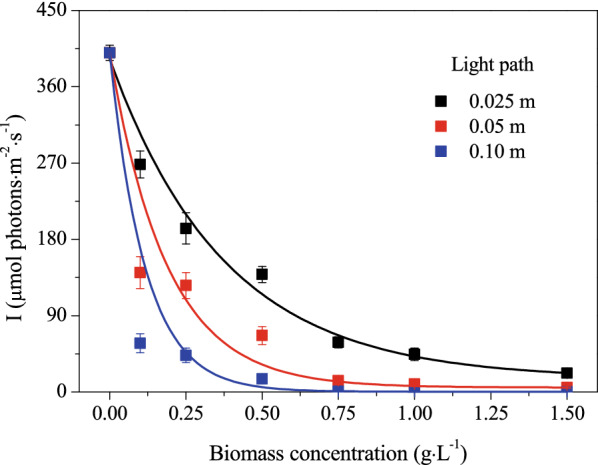
Variation of local light intensity with microalgae concentration and light path. The measurements were carried out in triplicate. Data shown as mean ± standard deviation, n = 3

Light distribution in the *D. salina* suspension with incident light intensity of 400 µmol photons·m^−2^·s^−1^ is shown in Fig. 1. For all lengths of light path, as the PBR was well-mixed, local light intensity attenuated exponentially with the increase of cell concentration, and longer light paths displayed much faster light attenuation. At 0.25 g·L^−1^ cell concentration, the local light intensity at 0.025, 0.05, 0.10 light paths, respectively, decreased by 51.8%, 68.60% and 89.2% of the incident light intensity. While at 0.5 g·L^−1^ cell concentration, corresponding local light intensities were, respectively, reduced by 65.34, 83.36 and 96.17%. At 1.50 g·L^−1^ algae concentration, the light intensities of 22.34, 5.31 and 0.30 µmol photons·m^−2^·s^−1^ were measured at 0.025 m, 0.05 m, and 0.10 m light paths, respectively, holding only 0.08–5.6% of incident light intensity.

**Fig. 2:**
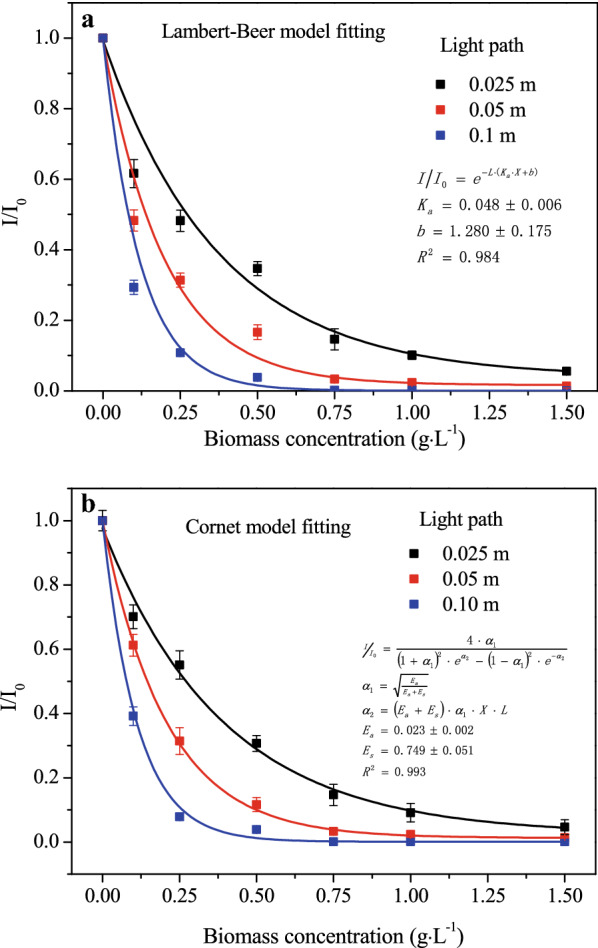
Experiment measured light attenuation and simulated values with **a** Lambert–Beer model and **b** Cornet model. In **a**, *K*_*a*_ is the extinction coefficient (m^2^·g^−1^), *X* is the microalgae concentration (g·L^−1^), *b* is the fitting constant (m^−1^). In **b**, *E*_*a*_ is the mass absorption coefficient (m^2^·g^−1^), and *E*_*s*_ is the mass scattering coefficient (m^2^·g^−1^). Lines are simulation results and points are experimental measurements

Figure 2 demonstrates the variation of transmittance (*I/I*_*0*_) with different cell concentrations at varied light paths. The results show that at low cell concentrations (˂ 0.75 g·L^−1^) light transmittance reduced significantly with increasing cell concentrations. When cell concentration was higher than 0.75 g·L^−1^, however, *I/I*_*0*_ leveled off at 0.1 for 0.025 m light path and almost zeroed at 0.05 m and 0.10 m light paths.

As evidenced by Fig. 2, the parameters calculated in this study were in good accordance with the classical Lambert–Beer model (*K*_*a*_ = 0.08 ± 0.006 m^2^·g^−1^, *b* = 1.280 ± 0.175 m^−1^, *R*^*2*^ = 0.984) and Cornet model (*E*_*a*_ = 0.023 ± 0.002 m^2^·g^−1^, *E*_*s*_ = 0.749 ± 0.051 m^2^·g^−1^, *R*^*2*^ = 0.993) on light attenuation evaluation, and it fitted slightly better with the Cornet model. Light attenuation is thought to be due to light scattering as well as absorption by microalgal cells themselves and by photosynthetic and accessory pigments at specific wavelengths. In order to quantify the light availability inside the PBR, it is necessary to measure the extinction coefficient at different cellular β-carotene contents as well.

### Effect of β-carotene content on light attenuation

The impact of β-carotene content on the light attenuation in the *D. salina* culture was investigated based on the assumption that the size and shape of all microalgae cells were consistent. The parameters in the Lambert–Beer model and Cornet model were estimated using data at different β-carotene contents, and are displayed in Table 1. From Table 1, it was revealed that the extinction coefficient *K*_*a*_ in the Lambert–Beer model and the scattering coefficient *E*_*s*_ in the Cornet model were positively related to β-carotene content, whereas the absorption coefficient *E*_*a*_ was negatively related to β-carotene content. *K*_*a*_, *E*_*s*_ and *E*_*a*_ as a function of β-carotene content are, respectively, displayed in Eqs. (13)–(15):

13 $$ K_{a} = \, 0.0466 + 0.0029 \times X_{{\beta - {\text{carotene content}}}} \left( {R^{2} = \, 0.98, \, r \, = \, 0.99} \right), $$

14 $$ E_{s} = \, 0.70 + 0.06 \times X_{{\beta - {\text{carotene content}}}} \left( {R^{2} = \, 0.95, \, r \, = \, 0.98} \right), $$

15 $$ E_{a} = \, 0.02 + 0.05 \times {\text{EXP}}( - 2.62 \times X_{{\beta - {\text{carotene content}}}} )\left( {R^{2} = \, 0.90, \, r \, = \, - 0.82} \right), $$

**Table 1:** Variation of *K*_*a*_, *E*_*a*_ and *E*_*s*_ with different *D. salina* β-carotene contents

β-carotene content (%)	Lambert–Beer model	Cornet model	
	*K*_*a*_ (m^2^·g^−1^)	*E*_*a*_ (m^2^·g^−1^)	*E*_*s*_ (m^2^·g^−1^)
0.56	0.0478 ± 0.0142	0.0316 ± 0.0015	0. 718 ± 0.021
0.84	0.0493 ± 0.0132	0.0242 ± 0.0010	0.754 ± 0.017
1.21	0.0504 ± 0.0175	0.0213 ± 0.0036	0.757 ± 0.014
1.93	0.0519 ± 0.0216	0.0207 ± 0.0042	0.846 ± 0.018
2.88	0.0558 ± 0.0224	0.0196 ± 0.0026	0.883 ± 0.013
4.26	0.0587 ± 0.0144	0.0167 ± 0.0032	0.948 ± 0.060

**Fig. 3:**
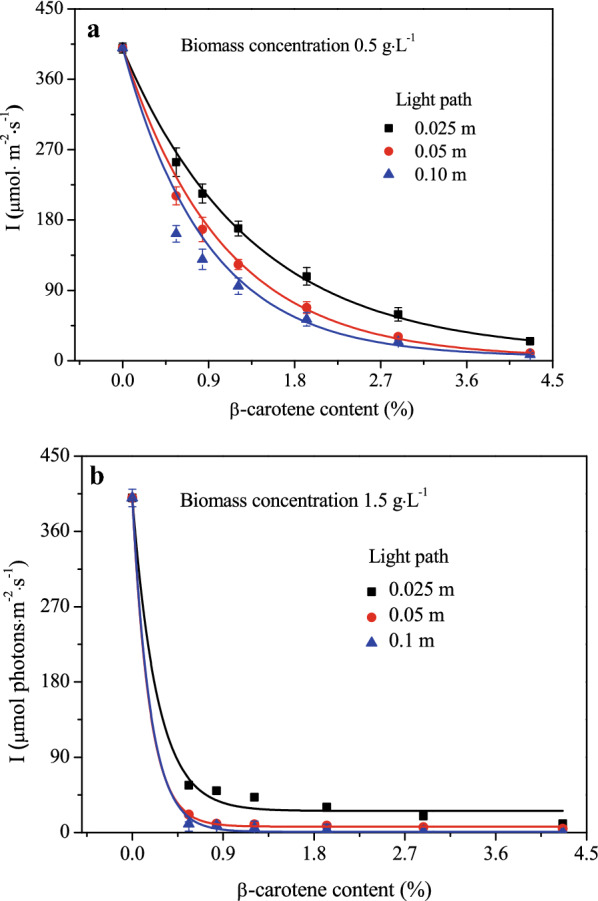
Variation of local light intensity with light path at different β-carotene contents using Cornet model. **a** Microalgae concentration of 0.5 g∙L^−1^, **b** microalgae concentration of 1.5 g∙L^−1^. Lines are simulation results and points are experimental measurements

Values are mean (± SD) of n = 3 cultivations per treatment, *K*_*a*_ is the extinction coefficient (m^2^·g^−1^), *E*_*a*_ is the mass absorption coefficient (m^2^·g^−1^), and *E*_*s*_ is the mass scattering coefficient (m^2^·g^−1^)

where X_β-carotene content_ is the dry weight content of β-carotene, while r is the Spearman's correlation coefficient, generally, there is a strong correlation between two events if the Spearman correlation coefficient exceeds 0.8 (Zhang et al. 2019), and R^2^ is the regression coefficient. Figure 3 displays the effect of β-carotene content on light attenuation under different biomass concentrations, using Cornet model, with relatively low (0.5 g∙L^−1^) and high (1.5 g∙L^−1^) biomass concentrations, respectively, investigated. It was revealed that β-carotene content had a greater influence on light attenuation at low biomass concentration (Fig. 3a), while such influence was not as obvious at high biomass concentration (Fig. 3b).

### The relationship between APRPC and β-carotene accumulation

**Fig. 4:**
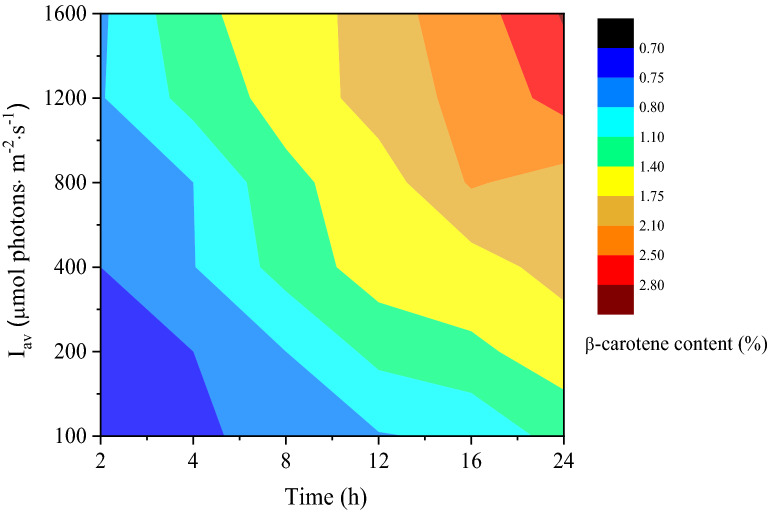
Effect of average irradiance and induction time on β-carotene content of *D. salina*. Red color represents high β-carotene content (%), and blue for low β-carotene content (%)

In order to calculate APRPC during β-carotene accumulation, it was necessary to analyze the effect of average irradiances (*I*_av_) and induction times on β-carotene accumulation. A contour-color fill plot of the variation in β-carotene content with various average irradiances and induction times is displayed in Fig. 4. From the results, it can be seen that β-carotene content increased with I_av_ and induction time. At 2 h induction time and 100 μmol photons·m^−2^·s^−1^ I_av_, low β-carotene content (0.70%) was obtained, implying that β-carotene accumulation had not started yet under this culture condition. When induction time was more than 8 h, and *I*_av_ was higher than 400 μmol photons·m^−2^·s^−1^, more than twice β-carotene content was obtained. The highest β-carotene content (2.83%) was reached at 24 h induction time and *I*_av_ of 1600 μmol photons·m^−2^·s^−1^.

**Fig. 5:**
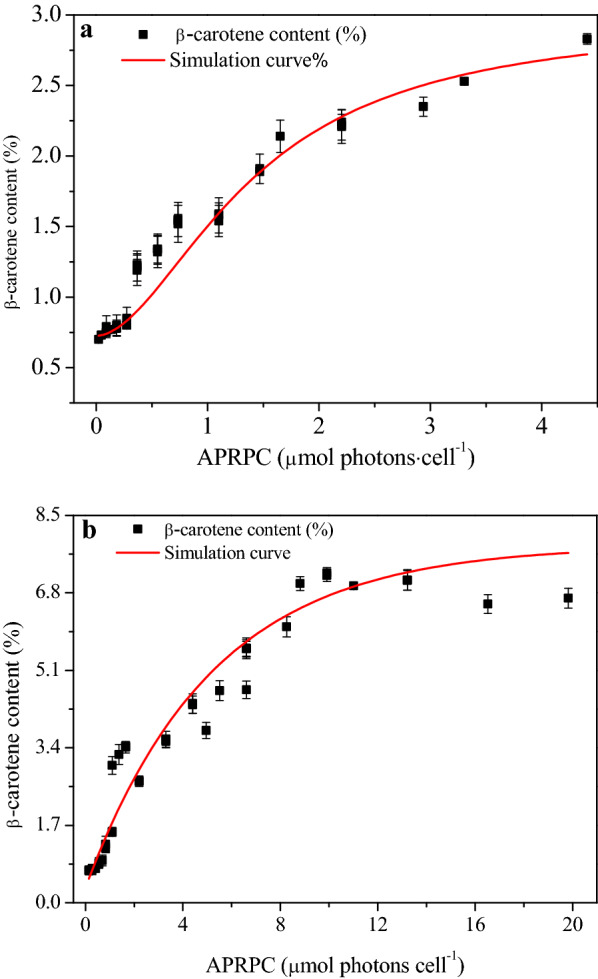
Correlation between β-carotene content (%) and the APRPC in *D. salina* cell. **a** The short-term (≤ 24 h) effect of APRPC on β-carotene content. **b** The long-term (24 h ≤ T ≤ 144 h) effect of APRPC on β-carotene content. Line is simulation result and points are experimental measurements. *APRPC* average number of photons received per cell

APRPC varied greatly under different induction times and irradiances (see Table 2), and was a function of β-carotene content for both short-term (≤ 24 h) and long-term (24 ~ 144 h) cultivation, as displayed in Fig. 5. The β-carotene content displayed positive correlations with APRPC but increased slowly at very low APRPC, implying that the APRPC was too low to induce substantial β-carotene accumulation in *D. salina.* Regarding both Table 2 and Fig. 4, it was found that when APRPC reached 0.7 µmol photons·cell^−1^, β-carotene content was twice original level. As displayed in Fig. 5a, within 24 h of induction, β-carotene content increased with APRPC. Thus, the quantitative relationship between APRPC and *D. salina* β-carotene accumulation was built. The data displayed in Fig. 5a are also helpful for optimal short-term β-carotene induction condition (average irradiance and induction time) estimation, which can benefit efficient β-carotene production on the whole.

**Table 2:** APRPC (µmol photons·cell^−1^) at different I_av_ and induction times

I_av_ (μmol photons·m^−2^·s^−1^)	Induction time (h)					
	2	4	8	12	16	24
100	0.02 ± 0.001	0.05 ± 0.002	0.09 ± 0.002	0.14 ± 0.002	0.18 ± 0.001	0.28 ± 0.002
200	0.05 ± 0.002	0.09 ± 0.002	0.18 ± 0.001	0.28 ± 0.002	0.37 ± 0.002	0.55 ± 0.002
400	0.09 ± 0.002	0.18 ± 0.001	0.36 ± 0.002	0.55 ± 0.002	0.73 ± 0.001	1.10 ± 0.030
800	0.18 ± 0.002	0.37 ± 0.002	0.73 ± 0.001	1.10 ± 0.030	1.47 ± 0.030	2.20 ± 0.012
1200	0.27 ± 0.001	0.55 ± 0.002	1.10 ± 0.002	1.65 ± 0.010	2.20 ± 0.012	3.30 ± 0.014
1600	0.37 ± 0.001	0.73 ± 0.001	1.47 ± 0.030	2.20 ± 0.012	2.94 ± 0.042	4.40 ± 0.024

Values are mean (± SD) of n = 3 cultivations per treatment

*APRPC* average number of photons received per cell

A long-term (24 ~ 144 h) light induction strategy was applied to get higher β-carotene content, with results displayed in Fig. 5b. The highest β-carotene content of 7.24% was observed at APRPC of 9.9 μmol photons·cell^−1^ at the end of cultivation, followed by 7.10% at 13.2 μmol photons·cell^−1^, and 7.0% at 11 μmol photons·cell^−1^, respectively. An increase in cellular chlorophyll content is commonly observed in strains grown under low APRPC, conversely, β-carotene content increase in cells exposed to high APRPC in order to minimize photo-oxidation processes (Fig.S2). The cellular β-carotene content is thus a main indicator of the light acclimation state of cells.

## Discussion

Previous studies indicated that the average irradiance was the key factor for carotenoids induction in microalgae, and have extensively used this parameter to describe light supply to PBRs (Fernandez et al. 1998; Grima et al. 1997, 1994; Zhang et al. 2016). However, average volume irradiation in the PBR was difficult to keep constant, due to the ever changing biomass concentration throughout the cultivation process, and sometimes accompanied by varied cellular pigments content as well. Therefore, selecting an appropriate illumination strategy and adjusting the culture conditions are crucial to achieve quantitative control for β-carotene production, and this would provide a more precise culture condition for quality control, instead of depending on the ever changing solar radiation. The average irradiance inside the PBRs in the present research was kept constant by feedback control of the incident irradiance according to the measured transmitted irradiance, biomass concentration, and β-carotene content during the whole induction period, and the ratio of average irradiance and biomass concentration was used to optimize APRPC, thus APRPC was controlled during the β-carotene accumulation induction stage. With this method, the effect of APRPC on β-carotene accumulation was investigated. Previous study calculated the carotenoid yield (mg·L^−1^) on absorbed light energy (mol^−1^ photons) (Fachet et al. 2016), but this is not enough to determine how many photons are required to trigger the accumulation of β-carotene. Kandilian et al. (Kandilian et al. 2014, 2019) proposed a concept of “MVERA” (mean volumetric rate of energy absorption, which is a function of incident irradiance and light attenuation in the PBR depending on biomass concentration and cellular pigment content) to quantify microalgal TAG accumulation and pigment synthesis, and MVERA is the number of photons absorbed by the cell per unit time. In this study, APRPC is the number of photons absorbed by the cell during the whole induction period, it is a function of average irradiance and biomass concentration in the PBR depending on cellular β-carotene content and light path, rather than the incident irradiance in the MVERA, so the number of photons absorbed by per cell to initiate β-carotene accumulation can be calculated more accurately by APRPC in this study.

The relationship between β-carotene content and light attenuation with different biomass concentrations in *D. salina* was also investigated in this study. The light attenuation in *D. salina* cells in present study was not consistent with previous studies (Garcia-Malea et al. 2006; Sheng et al. 2018), and revealed better explanation by Cornet model, rather than Lambert–Beer model, although Lambert–Beer model was successfully used in modeling other microalgae species such as *Phaeodactylum tricornutum* at low biomass concentration (Fernandez et al. 2000). Such discrepancy could be because the size of the *D. salina* cells was much bigger (15–20 μm at the β-carotene accumulation stage) comparing with 3–8 μm of *P. tricornutum* cells, thus the light scattered by microalgal cells could not be neglected. Also, the results obtained from this study were not in accordance with previous observation that decrease in carotenoids concentrations led to decrease in absorption coefficient (Kandilian et al. 2014). Actually, light absorbed by cells themselves was also considered in calculating the absorption coefficient. The scattering coefficient was greater than the absorption coefficient in this study, and this difference between both coefficients could be due to the great changes in cellular composition, as well as β-carotene content of the cells (Kandilian et al. 2019). It is noteworthy that scattering coefficients are greater than the absorption coefficients owing to high β-carotene contents in *D. salina* cells.

In this study, it was showed that APRPC can be used as an important parameter to accurately simulate and control β-carotene production in *D. salina* cultivation process. It was found that once APRPC reached 0.7 µmol photons cell^−1^, β-carotene accumulation was triggered, and it was saturated at 9.9 µmol photons cell^−1^. Considering the estimations that each microalgal cell needs 1 µmol$.4\times {10}^{-4}$ photons of light energy on average to maintain basic metabolism (Grima et al. 1997; Ogbonna and Tanaka 2000), this amount of energy is quite low compared with the 0.7 µmol photons cell^−1^ proposed by this study. According to previous study, 3.3–6.6 µmol photons of light energy would lead to photoinhibition in microalgae cells (Carvalho et al. 2011), which is much lower than 9.9 µmol photons cell^−1^ (the critical value to cause β-carotene accumulation saturation). This discrepancy may be due to the fact that synthesis of β-carotene requires more light energy than other secondary metabolites. Alternatively, it may be a response to acclimate to very high irradiances and is triggered by the generation of reactive oxygen species at high irradiances (Shaish et al. 1993). It has to be noted that, however, we have done this work at only one salinity, while the rate of β-carotene accumulation is not only light dependent, but also salinity dependent (Borowitzka et al. 1990) and the maximum content accumulated is also salinity dependent (Ben-Amotz and Avron 1983; Loeblich 1982). To overcome such limitation, the relationship between APRPC and β-carotene accumulation in *D. salina* under different salinities will be conducted in our future research.

APRPC can be used as an important parameter to be controlled in *D. salina* β-carotene production for both indoor and outdoor cultivation. Under laboratory conditions, artificial lights are used as the energy source for β-carotene production. As light quality is also important in carotenoid accumulation in *D. salina* (Xu and Harvey 2019a,^2020^; b), the average number of photons received per cell under a specific light spectrum should also be considered in the future, so that the red light effects and the fact that water preferentially absorbs red light could be accounted, thereby the precise control of β-carotene accumulation under any light intensity and light quality can be achieved. For massive scale cultivation, artificial light may also be used, thanks to the high value of natural β-carotene products. In this situation, the APRPC may be well controlled by adjusting average light intensity, light spectrum, illumination areas, cell density in the PBR, as well as the induction time. However, in most cases, sunlight is used for *D. salina* outdoor cultivation to save the production cost. In this situation, the input light intensity or light spectrum is not controllable, and it is difficult to maintain a constant APRPC. However, by adjusting the cell density, light path in the PBRs, as well as the induction time, similar level of APRPC during each day may be controlled. The cell density can be adjusted by diluting or concentrating the culture, and the light path can be adjusted by the depth of the culture.

It is notable that APRPC model established in this study may be used as an example for other carotenoids accumulation in other microalgae species, too: for example, the accumulation of astaxanthin in *H. pluvialis* and the accumulation of fucoxanthin by *P. tricornutum*. Although previous studies have made some attempts to control the average irradiance for astaxanthin induction in *H. pluvialis* (Sheng et al. 2018), the average irradiance inside the PBRs kept changing and the APRPC was not controlled in the process. With determining the parameters for astaxanthin in *H. pluvialis,* similar model may be developed, and this could realize the controllable accumulation of high-value carotenoids. Thus, this study provided a promising method to produce microalgal biomass with consistent carotenoids content, which is important for stable production and good quality control.

## Conclusion

Cornet model gave better prediction on calculating average light intensity in the process of β-carotene accumulation by *D. salina*. The APRPC can be controlled as a constant using the Algal Station system, despite of the ever changing cell density and carotenoids content in the cultivation. A minimum APRPC of 0.7 µmol photons·cell^−1^ was necessary to trigger significant *D. salina* β-carotene accumulation, and β-carotene content was saturated when APRPC reached 9.9 µmol photons cell^−1^. Biomass concentration and light path can be adjusted based on these APRPC characteristics in practical outdoor cultivation, so as to ensure that each cell gets a sufficient number of photons to accumulate a sufficient amount of β-carotene. Methods developed in this study can be used in other carotenoids production, such as astaxanthin by other microalgae species.

### Supplementary Information

**Additional file 1: Fig. S1**. The schematic overview of the Algal Station platform and light and temperature automatic control platform. 1: computer, running PC software; 2:pH displayer; 3: light intensity and mode regulator; 4: temperature regulator; 5: LED light source controller; 6: the control platform for temperature of the culture medium in photobioreactor; 7: Fv/Fm sensor; 8: RGB sensor; 9: OD sensor; 10: flat-plate photobioreactor; 11: outlight intensity sensor; 12: incident light intensity sensor. **Fig. S2**. The effect of APRPC on beta-carotene-to-chlorophyll ratio (car/chl). APRPC: average number of photons received per cell.

## Data Availability

Not applicable.
