# Supplementary material for: Quantitative analysis on photon numbers received per cell for triggering β-carotene accumulation in Dunaliella salina
Source: Bioresour Bioprocess. 2021 Oct 21;8(1):104. doi: 10.1186/s40643-021-00457-4 (PMC10992135; doi:10.1186/s40643-021-00457-4)
Supplement: Supplementary file 1 — Additional file 1: Fig. S1. The schematic overview of the Algal Station platform and light and temperature automatic control platform. 1: computer, running PC software; 2:pH displayer; 3: light intensity and mode regulator; 4: temperature regulator; 5: LED light source controller; 6: the control platform for temperature of the culture medium in photobioreactor; 7: Fv/Fm sensor; 8: RGB sensor; 9: OD sensor; 10: flat-plate photobioreactor; 11: outlight intensity sensor; 12: incident light intensity sensor. Fig. S2. The effect of APRPC on beta-carotene-to-chlorophyll ratio (car/chl). APRPC: average number of photons received per cell. [file 40643_2021_457_MOESM1_ESM.docx]

**Quantitative Analysis on Photon Numbers Received per Cell for Triggering β-Carotene Accumulation in *Dunaliella salina***

Yimei Xi^a,b^, Song Xue^b^, Xupeng Cao^c^, Zhanyou Chi^b^*, Jinghan Wang^b*^

^a^ Key Laboratory of Industrial Ecology and Environmental Engineering (Ministry of Education, China), School of Environmental Science and Technology, Dalian University of Technology, Dalian 116024, China

^b^ School of Bioengineering, Dalian University of Technology, Dalian 116024, China

^c^ Dalian Institute of Chemical Physics, Chinese Academy of Sciences, Dalian 16023, China

*Corresponding author:

Zhanyou Chi

Tel: +86-13234053986

E-mail: [chizhy@dlut.edu.cn](mailto:chizhy@dlut.edu.cn)

Jing-Han Wang

Tel: +86-18516696607

E-mail: [wangjinghan@dlut.edu.cn](mailto:wangjinghan@dlut.edu.cn)


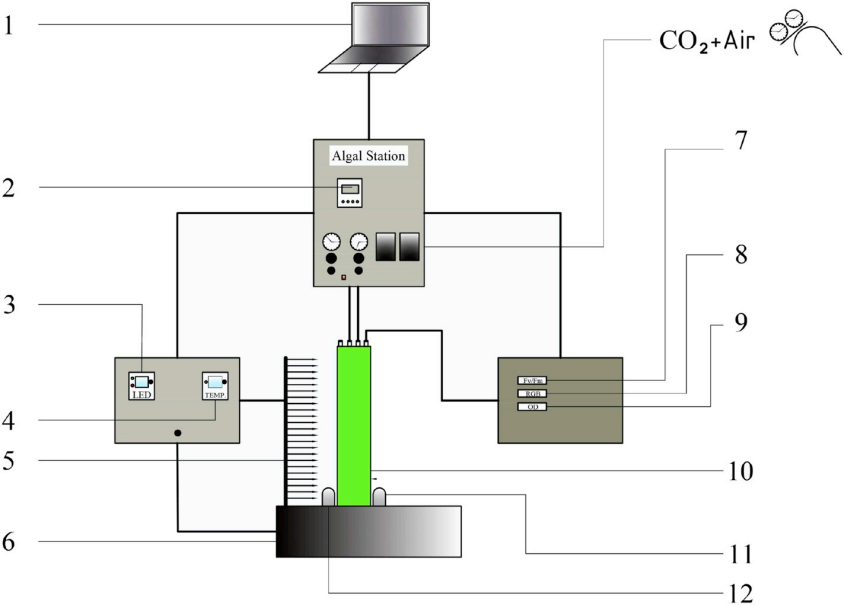


Fig.S1. The schematic overview of the Algal Station platform and light and temperature automatic control platform. 1: computer, running PC software; 2:pH displayer; 3: light intensity and mode regulator; 4: temperature regulator; 5: LED light source controller; 6: the control platform for temperature of the culture medium in photobioreactor; 7: *F_v_/F_m_* sensor; 8: RGB sensor; 9: OD sensor; 10: flat-plate photobioreactor; 11: outlight intensity sensor; 12: incident light intensity sensor.

Fig.S2. The effect of APRPC on beta-carotene-to-chlorophyll ratio (car/chl). APRPC: average number of photons received per cell.
